# Dysregulation of miR-146a: a causative factor in epilepsy pathogenesis, diagnosis, and prognosis

**DOI:** 10.3389/fneur.2023.1094709

**Published:** 2023-05-05

**Authors:** Shiqi Mao, Jinhan Wu, Jingkai Yan, Weijun Zhang, Feng Zhu

**Affiliations:** ^1^Department of Clinical Medicine, School of Medicine, Zhejiang University City College, Hangzhou, China; ^2^Department of Neurology, Zhejiang Provincial Hospital of Traditional Chinese Medicine, Hangzhou, China; ^3^Key Laboratory of Novel Targets and Drug Study for Neural Repair of Zhejiang Province, School of Medicine, Zhejiang University City College, Hangzhou, China

**Keywords:** miR-146a, epilepsy, NF-κB, inflammation, diagnosis, prognosis

## Abstract

miR-146a is an NF-κB-dependent miRNA that acts as an anti-inflammatory miRNA *via* the Toll-like receptor (TLR) pathway. miR-146a targets multiple genes and has been identified to directly or indirectly regulate processes other than inflammation, including intracellular Ca changes, apoptosis, oxidative stress, and neurodegeneration. miR-146a is an important regulator of gene expression in epilepsy development and progression. Furthermore, miR-146a-related single nucleotide polymorphisms (SNPs) and single nucleotide variants (SNVs) contribute to the genetic susceptibility to drug resistance and seizure severity in epilepsy patients. This study summarizes the abnormal expression patterns of miR-146a in different types and stages of epilepsy and its potential molecular regulation mechanism, indicating that miR-146a can be used as a novel biomarker for epilepsy diagnosis, prognosis, and treatment.

## 1. Introduction

Epilepsy is one of the most prevalent neurological disorders associated with multiple genetic and environmental factors. One-third of epilepsy patients suffer from relapsing drug-resistant epilepsy (DRE) (1). Recurrent seizures and unsynchronized neuronal firing in epilepsy majorly impact daily functioning and physical and mental health (2). Although inflammation, changes in synaptic strength, neuronal death, gliosis, and ion channel dysfunction have been reported as the underlying pathological processes in epilepsy, their pathogenesis remains incompletely understood. In addition, current clinical treatments cannot meet the needs of the patients (3). Current antiepileptic drugs (AEDs) provide only symptomatic epilepsy treatment, and there is no evidence that they have disease-modifying properties (4). Therefore, new epilepsy biomarkers and therapeutic targets are crucial for developing new diagnostic methods and personalized treatments.

**Figure 1:**
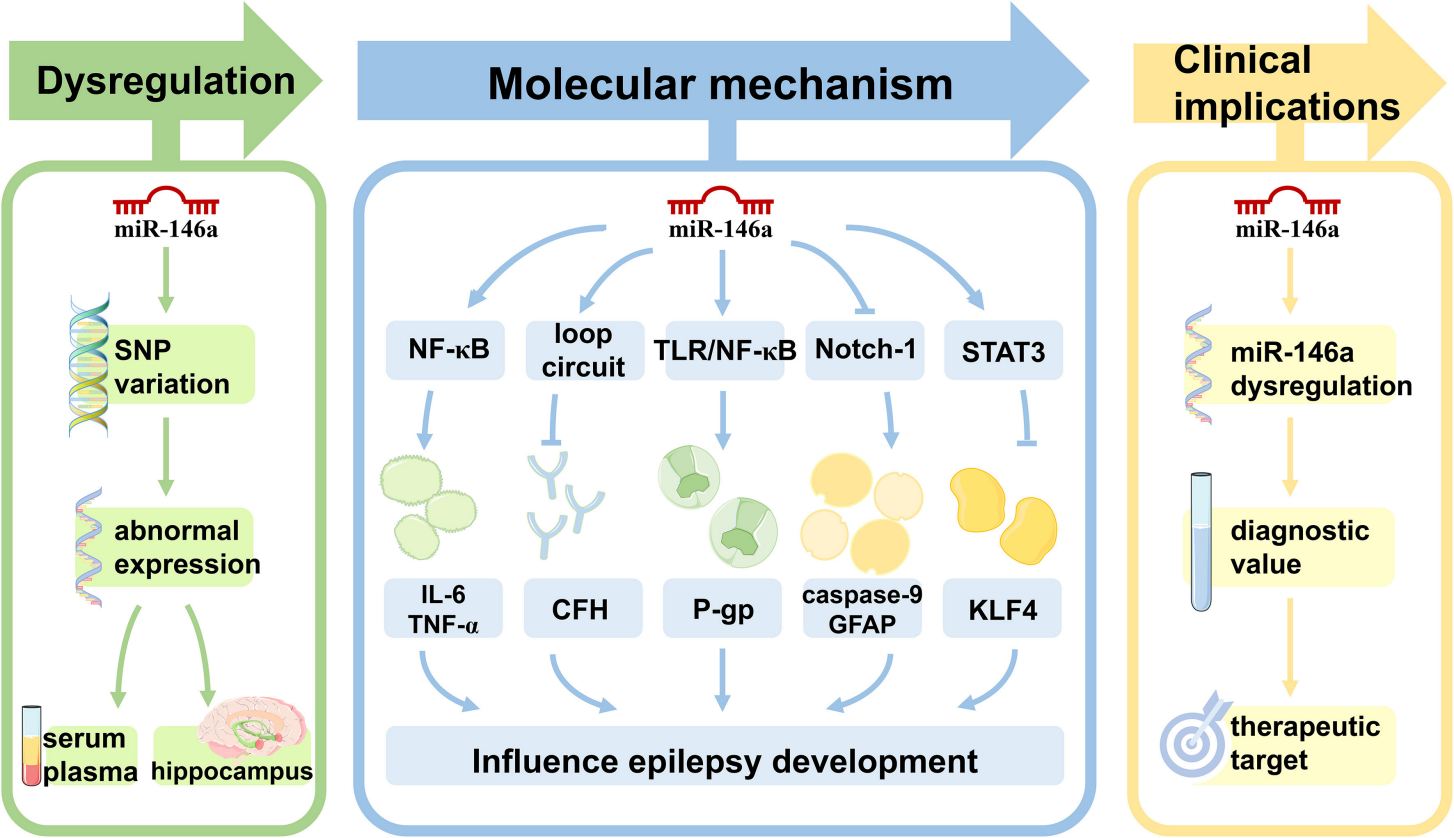
The role of miR-146a in epilepsy. Single nucleotide polymorphisms within miR-146a are significantly associated with the risk of epilepsy. In both animal models of epilepsy and human subjects, the expression pattern of miR-146a is aberrant in peripheral blood and/or brain. miR-146a is an NF-κB-dependent miRNA that acts as an anti-inflammatory miRNA *via* the Toll-like receptor pathway, targeting multiple genes to directly or indirectly regulate processes other than inflammation, including intracellular Ca changes, apoptosis, oxidative stress, and neurodegeneration in epilepsy development and progression. miR-146a may be used as a novel biomarker for epilepsy diagnosis, prognosis, and treatment.

miRNAs are a class of small non-coding RNAs−19–25 nucleotides in length (5). They bind to the 3'-untranslated regions of target mRNAs and inhibit the translation or degradation of target genes, thereby fine-tuning their function (6). As high-throughput sequencing technologies develop, emerging evidence suggests that dysregulation of miRNAs can affect epilepsy through both direct and indirect mechanisms (7). miRNAs have been implicated in synaptic structure and function, neurogenesis, neuronal migration, inflammation, transcription, and cell death in epilepsy pathophysiology (8). Basic and clinical studies have revealed that changes in miRNA expression at the brain level and changes in circulating miRNA levels in plasma are important diagnostic biomarkers of epilepsy (9). Acceptable sensitivity and specificity have validated high levels of miRNAs as biomarkers of epilepsy (10). Brain-enriched miR-146a is one of the most widely studied miRNAs in epilepsy. In this review, we focus on the role of miR-146a as an essential regulator of epilepsy development and progression and highlight its potential use as a therapeutic target and novel biomarker for epilepsy (Figure 1).

The human miR-146 family consists of two genes: miR-146a and miR-146b. miR-146a is in the long non-coding RNA host gene MIR3142HG on chromosome 5q33.3. miR-146a is highly conserved across species. Sequencing data collected from the miRBase database provided evidence that the miR-146a-5p strand is the biologically active “guide strand” (11). The primary transcript (pri-miR-146a) is under the control of a unique distinct promoter region enriched with CpG islands. It is a transcriptional regulatory sequence of more than 16kb upstream of the miR-146a locus (12). Several putative transcription factor binding sites have been identified, including at least one for CCAAT/enhancer-binding protein-β (C/EBP-β), one for interferon regulatory factor 3/7 (IRF3/7), and two for nuclear factor kappa-light-chain-enhancer of activated B cells (NF-κB) (13). Despite redundant target sequences among miR-146 family members, differences in their promoter regulatory sequences allow for the cell-specific regulation of miR-146a expression (14). miR-146 is expressed in neurons, astrocytes, and microglia (15). In addition, the long non-coding RNA nucleolar protein interacting with the FHA domain of pKi-67 (NIFK) and small nucleolar RNA host gene 16 (SNHG16) regulates miR-146a activity by base pairing and sequestering miRNAs away from their targets (16, 17).

miR-146a is central to neuroinflammation by modulating the NF-κB signaling pathway activity. It substantially impacts the homeostasis of immune and brain cells, neuronal identities, identity acquisition, and immune response regulation in the nervous system (18). Abnormal expression of miR-146a can cause the uncontrolled generation of neurotoxic inflammatory factors, such as IL-1, TNF-α, nitric oxide (NO), and hydrogen peroxide (H_2_O_2_), eventually leading to neuronal death and neurodegeneration (19, 20). Elevated expression of miR-146a in neural stem cells can lead to increased differentiation into neuronlike cells, higher neurite outgrowth, and branching (21). The two most important single nucleotide polymorphisms (SNPs) in miR-146a, rs2910164 and rs57095329, can affect the level of mature miR-146a, which is related to susceptibility to nervous system diseases (22).

## 2. miR-146a-associated variations and epilepsy

SNPs can affect the function or expression of a gene. Based on their location, miRNA-associated SNPs are classified into SNP sites on miRNA genes and their target genes (23).

**Figure 2:**
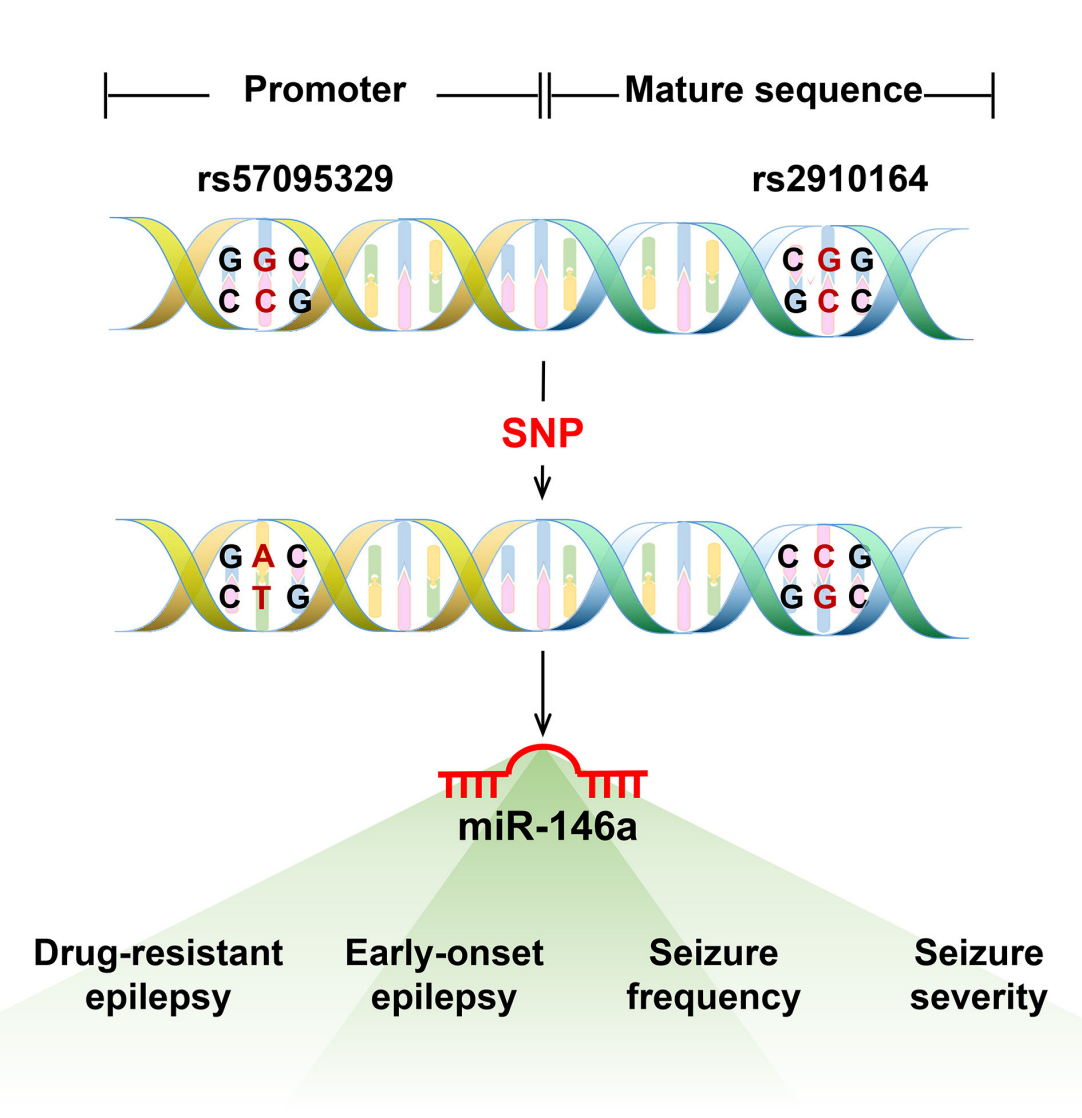
The most evaluated single nucleotide polymorphism (SNP) in miR-146a associated with epilepsy. The most evaluated SNPs associated with epilepsy susceptibility were n.60G > C (rs2910164) and n.-411A > G (rs57095329), located in the mature sequence and promoter region of miR-146a. The GC and CC genotypes of SNV rs2910164 in miR-146a are genetic susceptibility markers for DRE or early-onset epilepsy. The SNP rs57095329 in the miR-146a gene is associated with susceptibility to DRE and seizure frequency and also contributes to genetic susceptibility to DRE and seizure severity in children with epilepsy.

Single nucleotide polymorphisms in the sequences of miRNAs or their 3′-UTR target genes may affect the risk of epilepsy and expression of their target genes, thereby increasing disease susceptibility (23). The most evaluated SNPs associated with epilepsy susceptibility were n.60G > C (rs2910164) and n.-411A > G (rs57095329), which are located in the mature sequence and promoter region of miR-146a, respectively (8) (Figure 2). Boschiero et al. demonstrated that the GC genotype of SNV rs2910164 was associated with reduced miR-146a expression compared with the wild-type homozygous (GG) genotype and appeared to be associated with susceptibility to drug-resistant epilepsy (24). GC and CC genotypes of SNV rs2910164 in miR-146a are genetic susceptibility markers for DRE and early-onset epilepsy (8). Another study demonstrated that although a G-to-C substitution in SNP rs2910164 in miR-146a could influence miR-146a expression, it did not play a major role in TLE pathogenesis or some important clinical variables of TLE (25). Recent studies have found that SNP rs57095329 in the miR-146a gene is associated with susceptibility to DRE and seizure frequency (26) and contributes to genetic susceptibility to DRE and seizure severity in children with epilepsy (27).

## 3. Abnormal expression of miR-146a in epilepsy

In 2010, Aronica et al. first documented the altered expression pattern of miR-146a in epileptic rats and patients with temporal lobe epilepsy (28). They found that miR-146a expression levels were upregulated in astrocytes from temporal lobe epilepsy (TLE) patients with hippocampal sclerosis (HS), particularly in areas of neuronal destruction and gliosis. Since then, increasing evidence has depicted that miR-146a is abnormally expressed in human epilepsy patients and animal models (Tables 1, 2).

**Table 1:** Abnormal expression of miR-146a in epileptic patients.

**Seizure types**	**Control group**	**Level**	**miR-146a**	**Reference**
**Specimen in adult**				
PR-TLE with HS	Non-HS cases	Hippocampus	Upregulation	(28)
Epileptic patients	Non-epileptic patients	Hippocampus	Upregulation	(29)
FE and GE	Healthy controls	Serum	Upregulation	(30)
GS and PS	Healthy controls	Serum	Upregulation	(31)
TLE	Healthy controls	Serum	Upregulation	(32)
GGE	Healthy controls	Serum	Upregulation	(33)
Epileptic patients	Healthy controls	Plasma	Upregulation	(34)
FIAS	Favorable response to AEDs patients	Serum	Upregulation	(35)
**Specimen in children**				
DR-MTLE	No brain disease patients	Hippocampus	Upregulation	(36)
EEs	Healthy controls	Serum	Downregulation	(37)
IE	Healthy controls	Plasma	Upregulation	(38)

TLE, temporal lobe epilepsy; HS, hippocampal sclerosis; FE, focal epilepsy; GE, generalized epilepsy; GS, generalized seizures; PS, partial seizures; GGE, genetic generalized epilepsies; MTLE, mesial temporal lobe epilepsy; FIAS, focal impaired awareness seizures; EEs, epileptic encephalopathies; IE, idiopathic epilepsy; AED, antiepileptic Drugs; PR, pharmacologically refractory; DR, drug-resistant.

**Table 2:** Abnormal expression of miR-146a in animal models of epilepsy.

**Experimental epilepsy models**	**Control group**	**Time points after seizures**	**Level**	**miR-146a**	**Reference**
ES induced SE in rats	Implant but not stimulate	Latent phase	Hippocampus	Upregulation	(28)
		Chronic phase			
		Latent phase	Hippocampus	Upregulation	(39)
		Chronic phase			
		Chronic phase	Plasma		
Li–Pc induced SE in rats	NS	Chronic phase	The frontal cortex	Upregulation	(40)
		Latent phase	GCL; plasma	Upregulation	(29)
		Chronic phase			
		Acute phase	Hippocampus	Upregulation	(41)
		Chronic phase	Para-hippocampal cortex hippocampus	Upregulation	(42)
		Chronic phase	Hippocampus	Downregulation	(43)
Li–Pc induced SE in immature rats	No seizures	Latent phase	Hippocampus	Upregulation	(36)
		Chronic phase			

TLE, temporal lobe epilepsy; HS, hippocampal sclerosis; MTLE, mesial temporal lobe epilepsy; SE, status epilepticus; GCL, granule cell layer; KA, kainic acid; ES, electrical stimulation; Li, lithium; Pc, pilocarpine; NS, normal saline.

As illustrated in Table 1, the expression level of miR-146a was upregulated in the serum of patients with focal and generalized epilepsy (30, 31). Similar findings were observed in the frontal cortex of a pilocarpine-induced status epilepticus (SE) model (40). Furthermore, studies have shown displayed differences in the expression levels of miR-146a between various time points after seizures and in the control group (Table 2). Omran et al. found that in the hippocampus of a rat model of medial temporal lobe epilepsy (MTLE), miR-146a expression was the highest in the latent phase and lowest in the acute phase (36). Another study reported that miR-146a expression was significantly increased in the hippocampal granulosa cell layer (GCL) of TLE rats during the first spontaneous seizure, latency, and chronic phases after SE (29).

However, Song et al. demonstrated that miR-146a is downregulated in the hippocampus of rats with lithium-pilocarpine-induced chronic TLE (43). miR-146a downregulation has been reported in blood samples from children with epileptic encephalopathy (37). In addition, some studies have revealed that miRNA expression profiles in the brain and plasma are inconsistent. For example, in a study of epileptic rats, miR-146a expression was found to be upregulated in the brain 1 week after the onset of the disease. However, enhanced plasma expression was not observed until 3–4 months after chronic seizures (39).

## 4. The biological relevance of miR-146a in epilepsy

**Figure 3:**
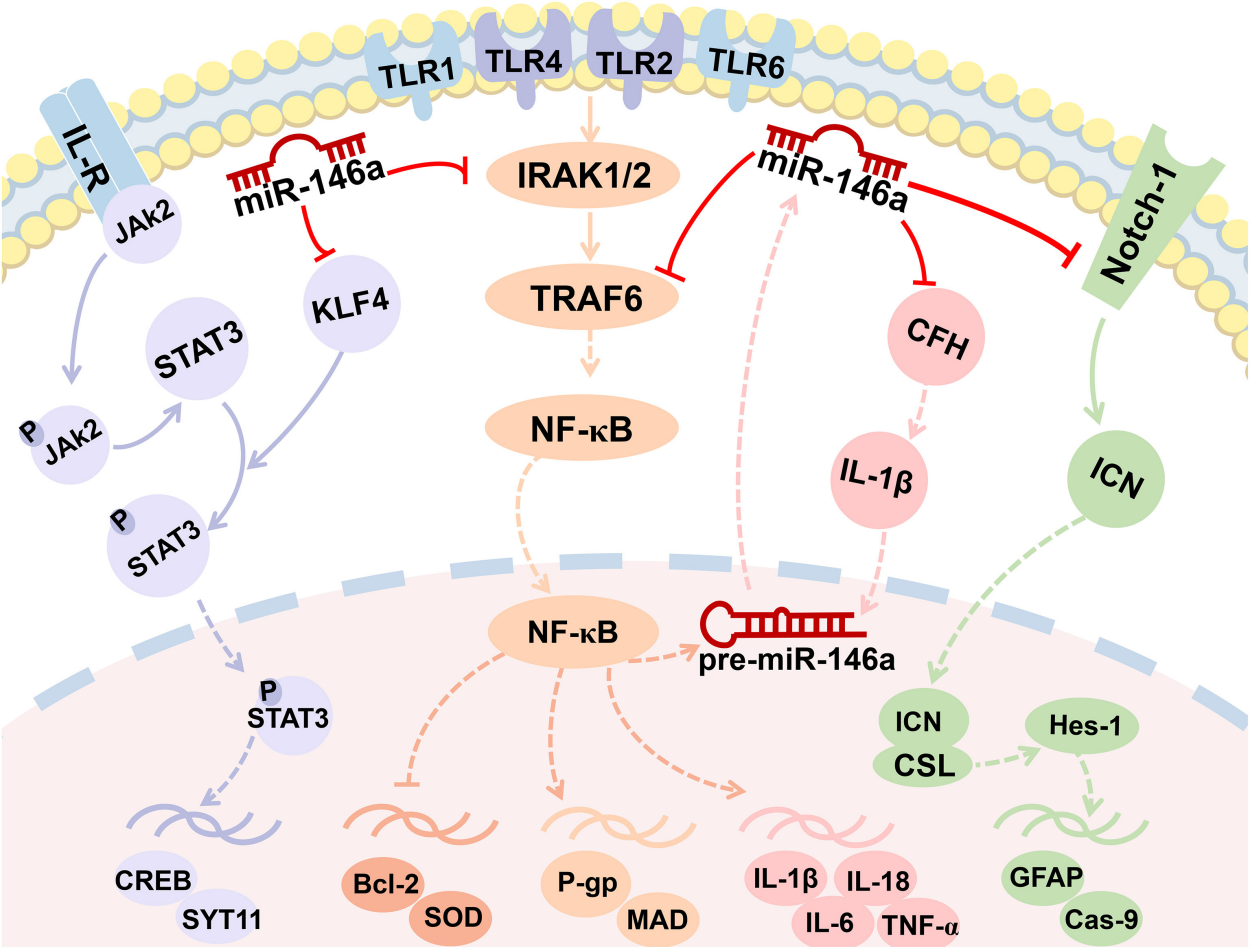
MiR-146a is involved in the regulation of multiple signaling pathways. miR-146a plays a crucial role in epileptogenesis as a negative feedback regulator of NF-κB signaling pathways. Enhanced miR-146a can boost IL-1β by downregulating the expression of CFH and forming a miR-146a-CFH-IL-1β loop circuit. The Notch-1 signaling pathway and JAK2/STAT3 signaling pathway were both the targets of miR-146a.

**Figure 4:**
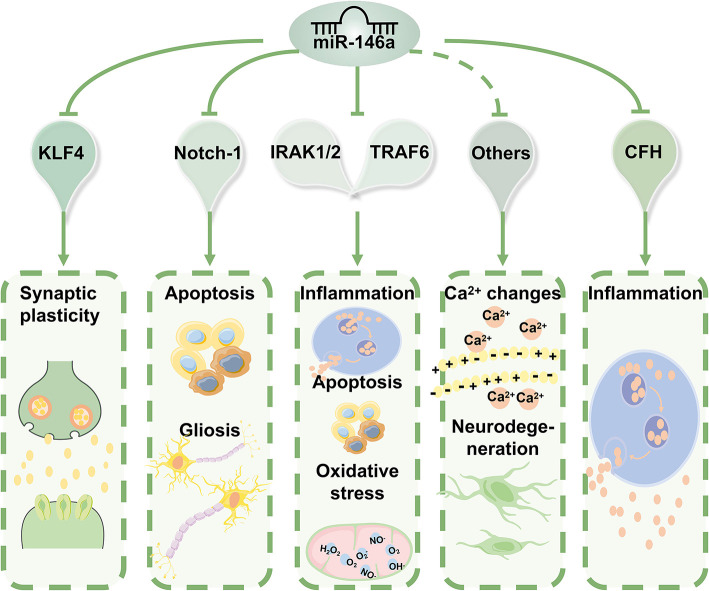
The molecular mechanisms of miR-146a in epilepsy. miR-146a acts as an anti-inflammatory miRNA can directly or indirectly regulate biological processes beyond inflammation, including intracellular Ca^2+^ changes, apoptosis, oxidative stress, and neurodegeneration. Based on the properties of its targets, miR-146a can have various biological implications in the pathophysiology and progression of epilepsy.

In addition to IRAK1/2 and TRAF6, the first reported targets of miR-146a, many other miR-146a targets that directly or indirectly regulate biological processes beyond inflammation, including intracellular Ca^2+^ changes, apoptosis, oxidative stress, and neurodegeneration, have been identified (Figures 3, 4) (44). miR-146a has been identified as a neuroimmune system mediator with major effects on immune and brain cell homeostasis, acquisition of neuronal identity, and modulation of immune responses in the nervous system (18).

Based on the properties of its target genes, miR-146a has various biological implications in the pathophysiology and progression of epilepsy. According to these findings, abnormal expression patterns and inefficient function of miR-146a are associated with epilepsy.

### 4.1. NF-κB and miR-146a in epilepsy

NF-κB is an essential regulator of inflammation and plays an important role in the occurrence and development of epilepsy (45). It is well known that miR-146a plays a crucial role in epileptogenesis as a negative feedback regulator of pro-inflammatory signaling pathways. Furthermore, seizure activity promotes downstream events activated by NF-κB, including inflammation and oxidative stress. This pathway also regulates neuronal excitability and seizure susceptibility (Figures 2, 3). Consequently, it appears that the role of miR-146a in epilepsy extends beyond that of neuroinflammation.

Most studies have revealed that miR-146a is significantly upregulated in epilepsy patients and experimental animal models. miR-146a silencing significantly reduced IL-1, IL-6, and IL-18 levels in the hippocampus of TLE rats and reduced neuronal damage in the CA1 and CA3 regions of the hippocampus (41). However, Wang et al. found opposite results by examining the effects of intracerebroventricular injection of miR-146a antagomir/agomir in an immature rat model of lithium/pilocarpine-induced status epilepticus (46). In the miR-146a antagomir injection group, the latency to generalized convulsions was shorter, the duration and degree of seizures were more severe, the expression level of miR-146a was clearly decreased, and IL-1β, TNF-α, TLR4, and NF-κB were significantly upregulated. The opposite was true for rats treated with miR-146a agomir.

### 4.2. Complement factor H (CFH) and miR-146a in epilepsy

Complement factor H is an essential and potent inhibitor of alternative complement activation (47). Researchers have confirmed that the persistence of complement activation contributes to a sustained inflammatory response and destabilizes neuronal networks in a TLE rat model (48). As displayed in Figure 3, in chronic TLE rats, enhanced miR-146a can boost IL-1β by downregulating the expression of CFH. Increased IL-1β further upregulated miR-146a expression, forming a miR-146a–CFH–IL-1β loop circuit that initiates an inflammatory cascade and perpetuates inflammation in TLE (Figures 3, 4) (49). However, another study, downregulating miR-146a by intracerebroventricular injection of antagomir-146a enhanced hippocampal expression of CFH and decreased seizure susceptibility in a TLE model (50).

### 4.3. P-glycoprotein and miR-146a in epilepsy

Activation of the TLR/NF-κB signaling pathway has recently been demonstrated to increase P-glycoprotein (P-gp) levels in the brains of rats with kainic acid-kindled epilepsy (Figure 3) (51). To the best of our knowledge, P-gp overexpression in epileptic patients and animal models is closely associated with antiepileptic drug resistance. Deng et al. found that miR-146a may decrease the level of P-gp expressed in the cerebral vessels of SE rats by downregulating the NF-κB signaling pathway (42). However, Zhang et al. reported that miR-146a gene silencing attenuates pathological changes and improves drug resistance in refractory epilepsy (52). According to this study, silencing of miR-146a downregulated P-gp expression and alleviated inflammation by regulating the high-mobility group box 1 protein (HMGB1)/TLR4/NF-κB signaling pathway.

### 4.4. Notch-1 and miR-146a in epilepsy

Notch signaling is well known for its requirement in the maintenance of neural stem/progenitor cells and the specification of glial cells (53, 54). Notch-1 has been identified as a target of miR-146a (Figures 3, 4) (55). The inhibitory role of miR-146a downregulation in neural damage in TLE is related to the inhibition of Notch signaling (41). It was found that miR-146a silencing inhibited apoptosis and gliosis in the hippocampus of TLE rats by downregulating the expression levels of caspase-9 and glial fibrillary acidic protein (GFAP) through Notch-1 signaling.

### 4.5. Signal transducer and activator of transcription 3 (STAT3) and miR-146a in epilepsy

Recent studies have highlighted the importance of miR-146a in epileptic synaptic plasticity. It was confirmed that miR-146a directly binds and downregulates the transcription factor Krüppel-like factor 4 (KLF4) to activate STAT3 and mediate epilepsy seizures by affecting synaptic plasticity in the pentylenetetrazole-kindling mouse model of epilepsy (Figures 3, 4) (56).

### 4.6. Other signaling pathways and miR-146a in epilepsy

Further molecular mechanisms underlying the role of miR-146a downregulation in cell apoptosis may be attributed to the mitigation of oxidative stress and inflammation (Figures 3, 4). miR-146a siRNA injection significantly decreased the levels of malondialdehyde (MDA), IL-1β, IL-6, and IL-18 but increased the level of superoxide dismutase (SOD) (41). Additionally, apoptosis is regulated by several signaling pathways, with the Bcl-2 protein family playing an important role (55). Zhang et al. revealed that treatment with a miR-146a inhibitor protected neurons from lithium/pilocarpine-induced toxicity and reversed lithium-pilocarpine-induced neuronal injury, partially by inhibiting the Bcl-2/Bax apoptotic pathway (57).

## 5. Molecular mechanism of miRNA-146a regulating target gene related to epilepsy

MicroRNAs regulate gene expression by targeting the 3′-UTR of mRNA, resulting in translational repression, mRNA degradation, or both. miR-146a was the first miRNA identified as NF-κB-dependent (58). Typically, miR-146a acts as an anti-inflammatory miRNA through the Toll-like receptor (TLR) pathway, regulating the expression of its target mRNAs, including interleukin 1 (IL-1) receptor-associated kinase-1/2 (IRAK1/2), tumor necrosis factor (TNF) receptor-associated factor 6 (TRAF6), and typical inflammatory modulators (NF-κB, TNF-α, IL-1β, and IL-6) (14). In response, the NF-κB interaction site on the miR-146a promoter allows interaction between NF-κB and AP1 to induce transcription at this site. It operates in a negative feedback loop by inhibiting two upstream NF-κB signaling components, IRAK1 and TRAF6 (Figure 3) (59).

Bioinformatics analysis indicated that miR-146a had overlapping target recognition sites within the CFH mRNA 3′-untranslated region (3′-UTR; 5′-TTTAGTATTAA-3′) (60). By binding to the putative sequence on the 3′-UTR of *cfh* mRNA and inhibiting both mRNA and protein expression of CFH, miR-146a may affect seizure susceptibility (50).

Online target gene prediction software (TargetScan) was used to identify a binding site for miRNA-146a in the 3′-UTR of Notch-1. Notch-1 was confirmed to be a target gene of miR-146a using a luciferase reporter assay (55). Notch signaling is required for epileptogenesis and neurogenesis (54). Once this pathway is blocked, neurogenesis is enhanced during the acute phase of epilepsy and neurogenesis during epileptogenesis is reduced (61).

A previous study identified pronounced effects of KLF4 knockout on promoting axon growth in developing neurons and facilitating regenerative axon growth after CNS injury (62). Ying et al. synthesized a mutated sequence of the binding site between miR-146a and KLF4 according to the prediction of the binding sites using the online software TargetScan and indicated that miR-146a targeted and inhibited KLF4 affecting epileptic seizures (56). Intriguingly, the circular RNA ANKS1B can act as a miRNA sequester for miR-146a to mediate post-transcriptional regulation of KLF4 expression (63).

## 6. Clinical implications of miR-146a in epilepsy

One of the potential clinical applications of miR-146a is the use of biofluid profiles for molecular diagnostics (64). Epilepsy diagnosis is primarily clinical, and evidence of the utility of circulating nucleic acids in epilepsy is underdeveloped relative to other diseases (65). Analyses performed on patient sera revealed that miR-146a is a suitable candidate as a circulating diagnostic molecule for several models of epilepsy (Table 3), distinguishing between epileptic patients and healthy subjects and even assessing disease risk and treatment responses.

**Table 3:** The diagnostic value of miR-146a in epilepsy.

**Number of samples**	**Seizure type**	**Healthy controls**	**Level**	**Diagnostic results**	**Diagnostic performance**			**Reference**
					**AUC**	**Sensitivity**	**Specificity**	
180	GS:57	90	Serum	Predict epilepsy	0.786	–	–	(30)
	PS: 33				0.887^*^	–	–	
289	GS:78	112	Serum	Improve the current diagnosis	0.784	81.9%	65.2%	(31)
	PS:39							
162	DR:76	–	Serum	Discriminate AED-resistant	0.64	–	–	(35)
	DRE:86							
146	GGE:79	67	Serum	Discriminate GGE patients	0.85^#^	80%^#^	73%^#^	(33)
50	IE:30	20	Plasma	Predict epilepsy in children	0.763	73.7%	60%	(38)
150	EP:80	70	Plasma	Complicate with cognitive dysfunction	0.808	87.8%	68.2%	(34)

AUC, Area under the receiver operating characteristic curve; TLE, temporal lobe epilepsy; GS, generalized seizures; PS, partial seizures; GGE, genetic generalized epilepsies; IE, idiopathic epilepsy; EP, epileptic patients; AED, antiepileptic drugs; DR, drug-sensitive; DRE, drug-resistant epilepsy;

* Combined with miR-106b;

# Combined with miR-155 and mir-132.

### 6.1. The diagnostic value of miR-146a in epilepsy

A pilot study found that miR-146a is a possible circulating diagnostic molecule (32). The miR-146a average expression in serum was 0.15 ± 0.11 in TLE patients compared to 0.07 ± 0.04 in healthy controls (*t*-test, *p*-value < 0.05). Moreover, there was a significant correlation between serum miR-146a levels and sex in the drug-resistant subjects (*p*-value < 0.05). Other studies' receiver-operating characteristic (ROC) curve and logistic regression analysis demonstrated that patients with elevated circulating miR-146a levels had a significantly increased risk of developing DRE (Table 3). Its effect was independent of temporal lobe sclerosis, epilepsy duration, family history, age at first seizure, body mass index (BMI), smoking, and gender. Decision curve analysis highlighted that evaluation of circulating miR-146a leads to superior clinical benefits for DRE prognosis and patient risk stratification (35). Elnady et al. evaluated circulating miR-146a as a diagnostic and prognostic biomarker in pediatric epilepsy patients and found that miR-146a expression levels were 14.65-fold higher in epileptic patients than in healthy controls (38). The ROC curve of serum miR-146a demonstrated a significant area under the curve (AUC) value of 0.763 for predicting epilepsy.

Recently, miR-146a was proposed as a diagnostic marker for genetically generalized epilepsy (GGE) in a panel of circulating miRNAs. The combined serum levels of miR-146a, miR-155, and miR-132 performed well as diagnostic biomarkers, discriminating GGE patients from controls with an AUC of 0.85, 80% specificity, and 73% sensitivity (33). Some researchers have found that miR-146a is strongly associated with cognitive dysfunction, anxiety, and depression in patients with epilepsy and reflects the severity of complications, indicating that it may serve as a potential biomarker for epilepsy (34).

### 6.2. The role of miR-146a as a therapeutic target in epilepsy

**Figure 5:**
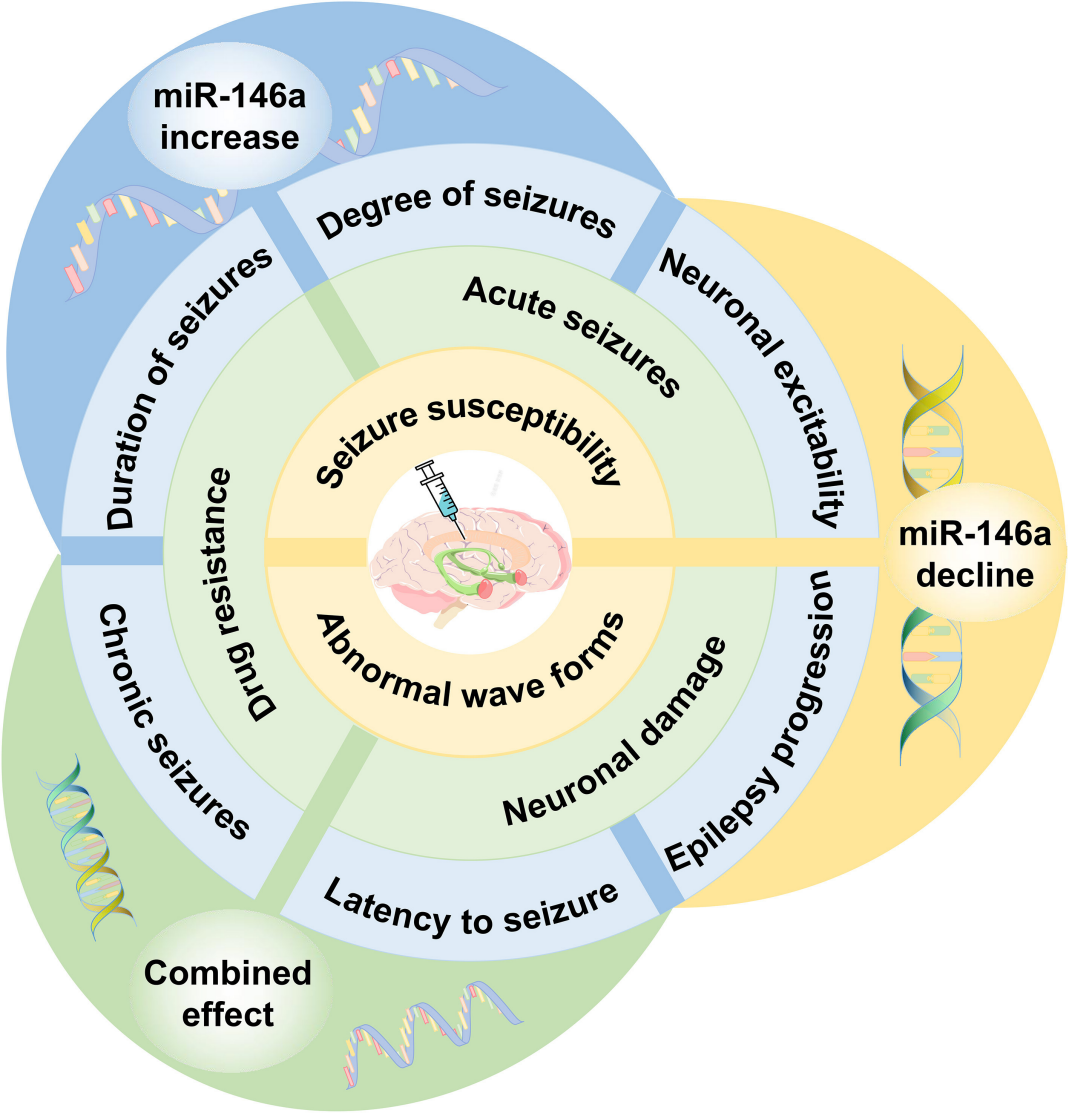
The effect of miR-146a as a therapeutic target in epilepsy. Regulating the expression of miR-146a to the optimal level can delay seizure onset, decrease seizure susceptibility, and improve drug resistance. miR-146a bears considerable potential for potential epilepsy therapeutic strategies.

Several researchers have focused on the therapeutic role of miR-146a. Some studies have attempted to regulate the expression of miR-146a and bring it to the optimal level by silencing or mimicking it with a synthetic antagomir or oligonucleotide mimic (Figure 5).

Iori et al. presented the first proof-of-concept data on disease modification using a transiently applied intervention after the onset of epilepsy (66). The results suggested that injection of miR-146a mimics reduced the number of seizures and the progression of the disease, including the postponement of disseminated seizures, improvement of structural hippocampal damage, and reduction in apoptosis of regional cells. Similarly, another study found that intracerebroventricular injection of miR-146a agomir inhibited NF-κB activity, alleviated neuroinflammation, and relieved seizures in SE rats (46). Intranasal administration of miR-146a mimics delayed seizure onset in a model of lithium-pilocarpine-induced status epilepticus (67). Meanwhile, studies have demonstrated that the injection of miR-146a mimics into the hippocampus of status epilepticus rats downregulated P-gp expression and improved drug resistance in refractory epilepsy (42).

However, other studies have reported contradictory results. Li et al. depicted that administration of miR-146a agomir in the hippocampal region upregulated the expression of miR-146a and increased abnormal waveforms in chronic TLE rat models (49). Downregulating miR-146a by intracerebroventricular injection of antagomir-146a enhanced hippocampal expression of CFH in the TLE model and decreased seizure susceptibility (50). Stereotactic injection of miR-146a antagomir reduced seizure scores in epileptic mice (56). Silencing of miR-146a downregulates P-gp expression and alleviates inflammation, which attenuates pathological changes and improves drug resistance in refractory epilepsy (52). According to a recent study, using miR-146a inhibitors to block pathogenic activation of NF-κB pathway in SE brain may have a neuroprotective function (57).

Overall, dynamic variations in miR-146a expression have been reported throughout distinct phases of epileptogenesis. These studies may promote miR-146a application as a biomarker and a novel therapeutic approach for epilepsy. The mechanism by which miR-146a functions as a biomarker in different stages of epilepsy remains unknown, and other variables that must be considered, such as age, comorbidities, and the nature of the induced injury, may affect the level of miR-146a expression. Some studies have revealed that miR-146a and other miRNA assays may have better sensitivity and specificity for predicting epilepsy. However, the altered biofluid profiles require further investigation. Furthermore, the levels of miR-146a expression must be measured separately in drug-resistant and drug-responsive patients because functions, targets, and processes of miR-146a in the development and pathogenesis of refractory epilepsy remain unclear, and it is not a predictor of drug response. Currently, miR-146a application as a biomarker of epilepsy is limited. Additional validation in large-scale studies is beneficial.

Previous studies have indicated that treatment with either anti-miR-146a or a miR-146a mimic ameliorates the latency, frequency, and duration of induced seizures in animal models of epilepsy, emphasizing the causality and reversibility of the effects of miR-146a in epilepsy. However, the rationale for the discrepancies between these studies remains unclear (21). A better understanding of the signaling pathways/feedback loops that can modulate the expression and action of miR-146a can help further therapeutic development. Extensive studies should be conducted in different epilepsy models, and other examples of the preclinical uses of miR-146a for treatment require further study.

## 7. Conclusion and prospects

In this review, we project a large summary of the role of miR-146a in brain functions, its expression in various models of epilepsy, and mechanistic links between their dysregulation in epileptic brain tissue and targeting for seizure control or disease modification. In the same line, we summarized the findings to date on circulating miRNA-146a levels as potential biomarker tools for diagnosing epilepsy and emphasized the application prospects of miR-146a in epilepsy therapy. It has been demonstrated that miR-146a, a crucial inflammation-related regulator, has advantages in therapeutic and diagnostic applications because of its ability to regulate message feedback circuits multi-directionally. Despite its potential, further exploration and verification are required to evaluate the different interfering factors in various types and stages of epilepsy.

Much work should be carried out before miR-146a can be used as a safe and effective therapeutic target for epilepsy, especially concerning its delivery methods and capacity for target specificity. There remains a major challenge in formulating medications that can regulate miR-146a expression, bring it to the optimal level, and improve drug development for targeted therapy. miRNA-targeting therapeutics must generally be administered *via* a route that circumvents the BBB or modifications that facilitate uptake *via* a systemic route (68). Intrathecal administration *via* lumbar puncture is invasive and inconvenient. Intranasal delivery may be a less invasive solution. Other safety-related risks arising from the difficulty in predicting the off-target effects of miRNA inhibition should be considered. As exosomes can cross the blood–brain barrier, exosomes originating in epileptogenic tissue may provide a means for non-invasive monitoring of seizure susceptibility and response to treatment (69). Because of the robust association between miR-146a and autoimmunity, miR-146a has a promising future for treating epilepsy based on intestinal microecosystems (70).

In conclusion, miR-146a, with the mentioned acts and potential, may be an ideal diagnostic marker and target/agent for epilepsy. Detailed information on the mechanisms and functional roles of miR-146a in epilepsy, which will be needed to develop novel candidate drugs for disease treatment, will require further investigation, and some of the unanswered questions and future directions should be considered.

## Author contributions

SM, JW, and JY: collected and analyzed the literature, drafted the figures, and wrote the manuscript. WZ and FZ: conceived and gave the final approval of the submitted version. All authors have read and agreed to the published version of the manuscript.
